# Retrovirus-Specificity of Regulatory T Cells Is Neither Present nor Required in Preventing Retrovirus-Induced Bone Marrow Immune Pathology

**DOI:** 10.1016/j.immuni.2008.09.016

**Published:** 2008-11-14

**Authors:** Inês Antunes, Mauro Tolaini, Adrien Kissenpfennig, Michihiro Iwashiro, Kagemasa Kuribayashi, Bernard Malissen, Kim Hasenkrug, George Kassiotis

**Affiliations:** 1 Divisions of Immunoregulation, The MRC National Institute for Medical Research, The Ridgeway, London NW7 1AA, UK; 2 Molecular Immunology, The MRC National Institute for Medical Research, The Ridgeway, London NW7 1AA, UK; 3 Laboratory of Persistent Viral Diseases, Rocky Mountain Laboratories, NIAID, NIH, Hamilton, MT 59840, USA; 4 Centre d'Immunologie de Marseille-Luminy, INSERM-CNRS-Université de la Méditerrannée, Case 906, 13288 Marseille Cedex 09, France; 5 Tazuke Kofukai Medical Research Institute, Osaka 530-8480, Japan; 6 Department of Oral and Maxillofacial Surgery, Faculty of Medicine, Kyoto University, Kyoto 606-8507, Japan

**Keywords:** CELLIMMUNO

## Abstract

Chronic viral infections of the hematopoietic system are associated with bone marrow dysfunction, to which both virus-mediated and immune-mediated effects may contribute. Using unresolving noncytopathic Friend virus (FV) infection in mice, we showed that unregulated CD4^+^ T cell response to FV caused IFN-γ-mediated bone marrow pathology and anemia. Importantly, bone marrow pathology was triggered by relative insufficiency in regulatory T (Treg) cells and was prevented by added Treg cells, which suppressed the local IFN-γ production by FV-specific CD4^+^ T cells. We further showed that the T cell receptor (TCR) repertoire of transgenic Treg cells expressing the β chain of an FV-specific TCR was virtually devoid of FV-specific clones. Moreover, anemia induction by virus-specific CD4^+^ T cells was efficiently suppressed by virus-nonspecific Treg cells. Thus, sufficient numbers of polyclonal Treg cells may provide substantial protection against bone marrow pathology in chronic viral infections.

## Introduction

A cardinal feature of adaptive immunity is that its response to infection is proportional to the pathogen load and antigen presentation, which regulate the expansion and contraction of antigen-specific lymphocytes (Zinkernagel and Hengartner, 2001). An effective immune response, which leads to pathogen control or clearance, is therefore self-limiting. Conversely, strong and lasting immune responses, which are ineffective in pathogen clearance, may be damaging to the host (Zinkernagel and Hengartner, 2001). Excessive or ineffective immune responses are characteristic of certain chronic viral infections, and their control is essential for the prevention of host tissue damage (Welsh and Selin, 2002; Zinkernagel, 2002; Guidotti and Chisari, 2006). Thus, several different mechanisms may operate, separately or in concert, to limit immune pathology. Exposure of T cells to high and persistent antigen loads has been shown to result in functional exhaustion or physical loss of antigen-specific T cells (Moskophidis et al., 1993; Zajac et al., 1998). The reactivity of antigen-specific T cells can also be actively regulated by potent suppressive mediators or specialized cell types, such as naturally occurring regulatory T (Treg) cells (Mills, 2004; Rouse et al., 2006; Belkaid, 2007).

Although many different cell types have been reported to exert inducible immune-suppressive activity (Shevach, 2006), Treg cells appear to be the largest subset in the body with naturally endowed suppressive activity (Sakaguchi, 2004; Kronenberg and Rudensky, 2005; Fontenot and Rudensky, 2005; Picca et al., 2006). Treg cells make up a substantial proportion (∼10%) of MHC class II-restricted CD4^+^ T cells and although they share many molecular markers with conventional CD4^+^ T cells, they also express subset-defining markers, such as forkhead box P3 (FoxP3), which directs Treg cell development and function (Sakaguchi, 2004; Fontenot and Rudensky, 2005). Acquisition of FoxP3 expression and phenotypic and functional conversion to Treg cells has been demonstrated in conventional naive CD4^+^ T cells, stimulated under predefined in vitro or in vivo conditions (Shevach, 2006). However, the Treg cell population found naturally in unmanipulated mice is almost exclusively of thymic origin (Sakaguchi, 2004; Fontenot and Rudensky, 2005; Pacholczyk et al., 2006), indicating that peripheral conversion of naive CD4^+^ T cells is very rare in steady-state conditions.

Similarly to conventional CD4^+^ T cells, in which induction of effector function requires antigenic stimulation, stimulation through the T cell receptor (TCR) is also considered necessary to induce suppressive function in Treg cells (Sakaguchi, 2004; von Boehmer, 2005). However, Treg cell-mediated suppression, once induced, can be directed toward T cells of unrelated specificity and is therefore antigen nonspecific (Thornton and Shevach, 2000; von Boehmer, 2005). Elucidation of the antigenic repertoire of Treg cells has been a matter of debate (Fontenot and Rudensky, 2005; Picca et al., 2006). Analyses of TCR repertoires in mice have indicated that, in comparison with naive CD4^+^ T cells, the TCR repertoire of Treg cells is of equal or even higher diversity, with a substantial number of clones found uniquely in the Treg cell pool (Hsieh et al., 2004; Pacholczyk et al., 2006; Wong et al., 2007). Although there are notable exceptions (van Santen et al., 2004; Pacholczyk et al., 2007), most observations support a model in which relatively high-affinity recognition of self-peptide-MHC II complexes favors thymic development of Treg cells and skews their TCR repertoire toward recognition of self-peptides (Hsieh et al., 2004; Sakaguchi, 2004; Fontenot and Rudensky, 2005; Kronenberg and Rudensky, 2005; Picca et al., 2006). However, despite their importance for immune regulation, the nature of self-peptides recognized by Treg cells or the degree of their reactivity to foreign peptides is still unclear.

We have developed a system to study the pathological consequences of the T helper cell response to unresolving infection with Friend virus (FV), a murine retroviral complex, which causes persistent infection of the hematopoietic system (Hasenkrug and Chesebro, 1997). We showed that exuberant virus-specific CD4^+^ T cell response to FV infection resulted in bone marrow immune pathology and anemia, the severity of which crucially depended on the balance between FV-specific pathogenic CD4^+^ T cells and Treg cells. Importantly, pathogenic CD4^+^ T cells and Treg cells differed dramatically in their requirements for direct recognition of viral antigens. Analysis of mice, in which transgenic expression of the TCRβ chain from a FV-specific CD4^+^ T cell clone created a polyclonal TCR repertoire with increased frequency of FV-specific CD4^+^ T cells, indicated that the TCR repertoire of naturally occurring Treg cells in virus-naive mice was virtually devoid of FV-specific clones. Nevertheless, bone marrow pathology driven by FV-specific TCRβ-transgenic CD4^+^ T cells was efficiently suppressed by virus-nonspecific Treg cells. Thus, virus-reactivity was neither detected in Treg cells nor required for their suppressive function.

## Results

### TCRβ-Transgenic Mice with Increased FV-Specific T Helper Precursor Frequency

**Figure 1:**
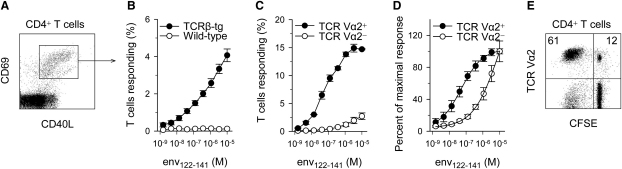
T Cell Reactivity to env_122–141_ and TCR Vα2 Usage in EF4.1 TCRβ-Transgenic Mice (A) Flow-cytometric example of CD69 and CD40L induction in CD4^+^ EF4.1 TCRβ-transgenic T cells 18 hr after stimulation with the env_122–141_ peptide. (B) Mean (±SEM) percentage of responding cells in total CD4^+^ T cells from EF4.1 TCRβ-transgenic or wild-type control mice 18 hr after stimulation. (C) Mean (±SEM) percentage of responding cells in gated TCR Vα2^+^ or Vα2^−^ CD4^+^ T cells from EF4.1 TCRβ-transgenic 18 hr after stimulation. (D) Mean (±SEM) percentage of responding cells, plotted as a fraction of the maximal response, in gated TCR Vα2^+^ or Vα2^−^ CD4^+^ T cells from EF4.1 TCRβ-transgenic 18 hr after stimulation. (E) TCR Vα2^+^ T cells in responding (CFSE^−^) or nonresponding (CFSE^+^) CD4^+^ T cells from EF4.1 TCRβ-transgenic 3 days after stimulation. Values in (A)–(E) are representative of five independent experiments.

To study the response of virus-specific T helper cells to retroviral infection, we have generated mice, here referred to as EF4.1, transgenically expressing the TCRβ chain of a TCR specific to an MHC class II-presented epitope of the surface (SU) product of the F-MuLV envelope (*env*) gene (Iwashiro et al., 1993). The transgenic TCRβ chain is able to associate with endogenous TCRα chains creating a polyclonal repertoire. EF4.1 TCRβ-transgenic CD4^+^ T cells responded to in vitro stimulation by an env 122–141 peptide (env_122–141_), by upregulating CD69 and CD40L (Figures 1A and 1B). This response was linear over a 4-log_10_ range of env_122–141_ concentration, suggesting a multiclonal composition reaching on average a maximum of 4% env-specific T cells in total CD4^+^ T cells (Figure 1B). We next examined the expression of particular endogenous TCR Vα families in responding EF4.1 TCRβ-transgenic CD4^+^ T cells and identified TCR Vα2 as one that conferred specificity and higher than average avidity for env_122-141_ (Figures 1C and 1D). Compared with their TCR Vα2^-^ counterparts, TCR Vα2^+^ EF4.1 TCRβ-transgenic CD4^+^ T cells contained a higher frequency of env-specific T cells (3% and 15%, respectively) (Figure 1C). Furthermore, Vα2^+^ env-specific CD4^+^ T cells were >30-fold more sensitive to env_122–141_ than their Vα2^−^ counterparts (ED_50_ = 0.04 μM and > 1.24 μM env_122–141_, respectively) (Figure 1D). As a result of differences in frequency and functional avidity for env_122-141_ between virus-naive TCR Vα2^+^ and Vα2^−^ env-specific T cells, TCR Vα2^+^ T cells, which normally represent 12%–14% of all CD4^+^ T cells in C57BL/6 (B6) mice, were highly enriched in the responding population (40%–60% TCR Vα2^+^) (Figure 1E).

### T Helper Cell Response to Retroviral Infection Results in Bone Marrow Pathology

**Figure 2:**
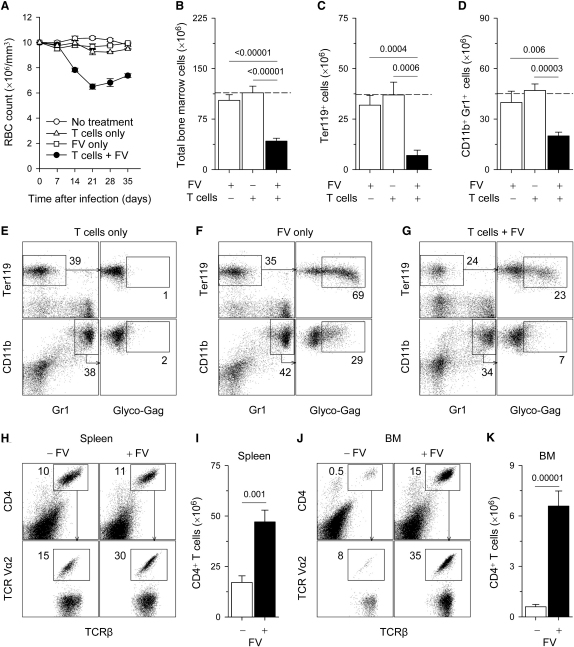
Continuous T Helper Cell Response to Unresolving FV Infection Causes Bone Marrow Pathology (A) Changes in RBC counts in *Rag1*^−/−^ mice, which were infected with FV (FV only) or received EF4.1 TCRβ-transgenic CD4^+^ T cells (T cells only), or both (T cells + FV). Values represent the mean (±SEM) of 8–18 mice per group per time point, analyzed in more than three independent experiments. p < 0.001 between “T cells + FV” group and all other groups for days 14–35. (B–D) Total bone marrow cellularity (B) and numbers of Ter119^+^ erythroid precursor cells (C) and CD11b^+^Gr1^+^ bone marrow cells (D) in the same recipient mice. The dashed line represents the average cell numbers of untreated *Rag1*^−/−^ mice. Values are the mean (±SEM) of 5–13 mice per group on day 21. Numbers within the graphs denote the p values. (E–G) Percentages of uninfected and FV-infected (glyco-Gag^+^) Ter119^+^ and CD11b^+^Gr1^+^ cells in the bone marrow of the same groups of mice described in (A). Numbers within the plots represent the percentage of positive cells are the average of more than five mice per group analyzed on day 21. (H–K) Percentage (H and J) and absolute numbers (I and K) of CD4^+^ T cells and percentage of TCR Vα2^+^ T cells in total CD4^+^ T cells (H and J) in the spleen (H and I) or the bone marrow (J and K) of uninfected (−FV) or FV-infected (+FV) recipients of EF4.1 TCRβ-transgenic CD4^+^ T cells. Values are representative of 5–8 mice per group analyzed on day 21.

To follow the immunopathological consequences of a continuous FV-specific T helper cell response, we adoptively transferred EF4.1 TCRβ-transgenic CD4^+^ T cells, containing FV-specific clones, into FV-infected immunodeficient *Rag1*^−/−^ hosts, in which the absence of CD8^+^ T cells or B cells prevents virus clearance or control. Starting from the second week of transfer, recipient mice developed symptoms of anemia reflected in red blood cell (RBC) counts in peripheral blood (Figure 2A). Anemia peaked on day 21 after adoptive transfer of EF4.1 TCRβ-transgenic CD4^+^ T cells and remained chronic with only modest recovery at later time points (Figure 2A). In contrast to concurrent transfer of EF4.1 TCRβ-transgenic CD4^+^ T cells and FV infection, neither EF4.1 TCRβ-transgenic CD4^+^ T cell transfer nor FV infection alone produced any substantial signs of anemia in the recipient mice in the time frame of these experiments (Figure 2A). These results suggest that anemia in this setting is a pathological consequent of the T helper response to FV infection, rather than FV infection itself.

To determine whether anemia was the result of reduced bone marrow function, we examined the cellularity and composition of the bone marrow in anemic mice. Neither FV infection nor EF4.1 TCRβ-transgenic CD4^+^ T cell transfer had any appreciable effect on bone marrow cellularity (Figure 2B–2D) or composition (Figure 2E–2G). In contrast, EF4.1 TCRβ-transgenic CD4^+^ T cell transfer in FV-infected recipients caused significant reduction in total bone marrow cell numbers (Figure 2B). This effect was more pronounced in Ter119^+^ erythroid precursors (Figure 2C), but also observed in the heterogeneous population of CD11b^+^Gr1^+^ myeloid precursors (Figure 2D), which together represent ∼75% of bone marrow cells in *Rag1*^−/−^ mice. Furthermore, EF4.1 TCRβ-transgenic CD4^+^ T cell transfer in FV-infected recipients was associated with significant functional reduction in hematopoietic colony-forming cells (Figures S1A and S1B available online) and histological hypocellularity with pronounced reduction in hyperchromatic normoblasts (Figure S1C), consistent with generalized myelosuppression. In FV-infected mice, two-thirds and one-third of Ter119^+^ and CD11b^+^Gr1^+^ bone marrow cells, respectively, were positive for the glycosylated product of the viral *gag* gene (glyco-Gag), an indicator of FV infection (Figure 2F). However, this was not associated with any bone marrow dysfunction, in line with the noncytopathic nature of FV infection. Transfer of FV-specific CD4^+^ T cells in FV-infected mice led to marked reduction in the number of infected bone marrow cells (Figure 2G), in agreement with the reported direct antiviral activity of FV-specific CD4^+^ T cells, mediated by IFN-γ (Iwashiro et al., 2001). However, substantial numbers of FV-infected cells remained present (Figure 2G), indicating incomplete virus control by FV-specific CD4^+^ T cells in the absence of CD8^+^ T cells and B cells.

Expansion of transferred EF4.1 TCRβ-transgenic CD4^+^ T cells in the spleens of FV-infected *Rag1*^−/−^ hosts was only marginally higher than homeostatic expansion of the same cells in the spleens of uninfected *Rag1*^−/−^ hosts (Figures 2H and 2I). Interestingly, expansion in response to FV infection, but not homeostatic expansion, was associated with enrichment of CD4^+^ T cells in TCR Vα2^+^ clones (Figure 2H). In contrast to homeostatic expansion in the spleen, significant numbers of transferred EF4.1 TCRβ-transgenic CD4^+^ T cells were found in the bone marrow of *Rag1*^−/−^ hosts only in the presence of FV infection (Figures 2J and 2K). CD4^+^ T cells isolated from the bone marrow of FV-infected recipients were also highly enriched in TCR Vα2^+^ clones (Figure 2J). Together, these results support a model in which failure to contain noncytopathic FV infection in *Rag1*^−/−^ hosts attracted FV-specific CD4^+^ T cells to the bone marrow, a location from which they were normally excluded, ultimately leading to bone marrow pathology. In support of this model, FV infection was also found in the bone marrow of B cell-deficient mice, which lack FV-neutralizing antibodies, but not of wild-type control mice (Figure S2A). Furthermore, in contrast to wild-type mice, B cell-deficient mice experienced significant anemia during FV infection (Figure S2B). Because B cell-deficient mice were T cell replete, these results indicated that, first, the contribution of immunodeficient hosts to anemia development was to allow the spread of FV replication to the bone marrow, rather than to provide a state of T cell-lymphopenia, and second, both EF4.1 TCRβ-transgenic and wild-type host T cells were capable of mediating bone marrow pathology in this setting.

### Treg Cells Suppress FV-Induced Bone Marrow Pathology

**Figure 3:**
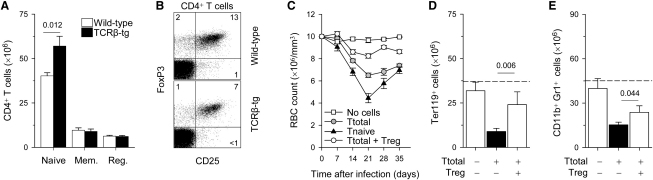
Suppression of Bone Marrow Pathology by Treg Cells in a Dose-Dependent Manner (A) Absolute numbers of naive CD44^lo^CD25^−^, memory (mem.) CD44^hi^CD25^−^ or regulatory (reg.) CD25^+^ CD4^+^ T cells in secondary lymphoid organs of EF4.1 TCRβ-transgenic (TCRβ-tg) and wild-type control mice. Values are the mean (±SEM) of six mice per group and numbers within the graphs denote the p value. (B) CD25 and FoxP3 staining in gated CD4^+^ T cells from EF4.1 TCRβ-transgenic (TCRβ-tg) and wild-type control mice. Numbers within the quadrants denote the percentages of positive cells and are representative of three mice per group. (C) Changes in RBC counts in FV-infected *Rag1*^−/−^ mice, which received total EF4.1 TCRβ-transgenic CD4^+^ T cells (Ttotal), FACS-purified Treg-depleted naive CD45RB^hi^CD25^−^ CD4^+^ T cells (Tnaive), or total CD4^+^ T cells together with FACS-purified CD25^+^ CD4^+^ T cells at 1:1 ratio (Ttotal + Treg). Values represent the mean (±SEM) of 8–12 mice per group per time point analyzed in three independent experiments. p = 0.0002 between “Ttotal” and “Ttotal + Treg,” and p = 0.00002 between “Ttotal” and “Tnaive,” on day 21. (D and E) Numbers of Ter119^+^ erythroid precursor cells (D) and CD11b^+^Gr1^+^ bone marrow cells (E) in FV-infected *Rag1*^−/−^ mice, which received EF4.1 TCRβ-transgenic CD4^+^ T cells, alone or together with EF4.1 TCRβ-transgenic Treg cells at 1:1 ratio. The dashed line represents the average cell numbers of untreated, uninfected *Rag1*^−/−^ mice. Values are the mean (±SEM) of 5–8 mice per group on day 21. Numbers within the graphs denote the p values.

The development of aplastic anemia in FV-infected *Rag1*^−/−^ recipients of EF4.1 TCRβ-transgenic CD4^+^ T cells highlighted the pathogenic potential of FV-specific clones. However, donor CD4^+^ T cells should also contain Treg cells, which would counteract the pathogenic effect of excessive T helper response to FV infection. We first confirmed that development of Treg cells was not perturbed in EF4.1 TCRβ-transgenic mice. As expected in mice transgenic for a TCR with bias for MHC class II recognition, there was an increase in absolute numbers of naive CD4^+^ T cells (Figure 3A). In both wild-type and EF4.1 TCRβ-transgenic CD4^+^ T cells, expression of CD25 overlapped with expression of Treg cell-specific transcription factor FoxP3 (Figure 3B) and the absolute numbers of Treg cells were comparable between the two groups of mice (Figure 3A). However, the percentage of Treg cells in total CD4^+^ T cells was reduced in EF4.1 TCRβ-transgenic mice, in comparison with wild-type mice, because of the increase in naive CD4^+^ T cells (Figures 3A and 3B).

To examine whether Treg cells in the transferred EF4.1 TCRβ-transgenic CD4^+^ T cell population were working against pathology-inducing FV-specific T cells, we compared transfers of total CD4^+^ T cells with transfers of Treg-depleted naive CD4^+^ T cells. RBC counts in recipients of naive CD4^+^CD45RB^hi^CD25^−^ EF4.1 TCRβ-transgenic T cells dropped significantly lower than in recipients of total CD4^+^ T cells, reaching their lowest value on day 21 after T cell transfer, which was followed by partial recovery to counts comparable to those in recipients of total CD4^+^ T cells by day 35 (Figure 3C). Thus, the overall severity of anemia was exacerbated by removal of Treg cells from the transferred CD4^+^ T cell population. We therefore reasoned that supplementing the total CD4^+^ T cell population with additional Treg cells would have a protective effect against bone marrow pathology. Indeed, addition of extra EF4.1 TCRβ-transgenic Treg cells led to substantial, albeit incomplete, suppression of anemia development (Figure 3C). Suppression of anemia by Treg cells was also accompanied by restoration of numbers of Ter119^+^ (Figure 3D) and CD11b^+^Gr1^+^ bone marrow cells (Figure 3E), indicating that Treg cells were acting by reducing bone marrow pathology. Thus, the severity of bone marrow pathology during the CD4^+^ T cell response to unresolving FV infection appeared to be directly proportional to the relative frequency of Treg and pathogenic T cells.

### Treg Cells Limit IFN-γ Production by Pathogenic T Cells

**Figure 4:**
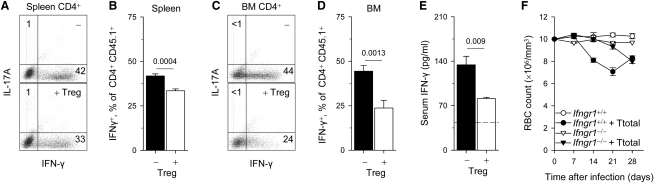
Treg-Mediated Suppression of Local IFN-γ Production by Conventional EF4.1 TCRβ-Transgenic CD4^+^ T Cells in the Bone Marrow (A–D) Percentage of IL-17A and IFN-γ producing cells in total CD45.1^+^ EF4.1 TCRβ-transgenic CD4^+^ T cells, isolated from the spleen (A and B) or the bone marrow (C and D) of FV-infected *Rag1*^−/−^ mice, which received total CD45.1^+^ EF4.1 TCRβ-transgenic CD4^+^ T cells, alone (−) or together (+ Treg) with CD45.2^+^ EF4.1 TCRβ-transgenic Treg cells at 1:1 ratio. Values are representative of six to nine mice per group, analyzed either on day 21 or day 35 with identical results. Numbers within the bar graphs and dot plots denote the p values and percentage of positive cells, respectively. The median fluorescence intensity (MFI) ± SEM of IFN-γ staining in C was “− Treg” = 160 ± 19; “+ Treg” = 105 ± 15; p = 0.0003, Student's t test, n = 6. (E) Serum IFN-γ concentration in FV-infected *Rag1*^−/−^ mice, which received total CD45.1^+^ EF4.1 TCRβ-transgenic CD4^+^ T cells, alone (−) or together (+ Treg) with CD45.2^+^ EF4.1 TCRβ-transgenic Treg cells at 1:1 ratio. Values represent the mean (±SEM) of four to five mice per group analyzed on day 35. The dashed line depicts the basal concentration of serum IFN-γ in unmanipulated *Rag1*^−/−^ mice. Numbers within the graph denote the p values. (F) Changes in RBC counts in *Rag1*^−/−^*Ifngr1*^+/+^ (*Ifngr1*^+/+^) or *Rag1*^−/−^*Ifngr1*^−/−^ (*Ifngr1*^−/−^) mice either infected with FV alone (*Ifngr1*^+/+^; *Ifngr1*^−/−^) or infected with FV and received CD4^+^ T cells (*Ifngr1*^+/+^ + T cells; *Ifngr1*^−/−^ + T cells). Values represent the mean (±SEM) of 12–15 mice per group per time point analyzed in three independent experiments. p < 0.0001 between “*Ifngr1*^+/+^ + T cells” and “*Ifngr1*^−/−^ + T cells,” on days 14 and 21.

To further investigate the mechanisms by which FV-induced bone marrow pathology was suppressed by Treg cells, we examined their effect on the expansion and effector function of FV-specific pathogenic T cells. Addition of EF4.1 TCRβ-transgenic Treg cells caused a 50% reduction in the expansion of CD4^+^ T cells in the spleens of FV-infected recipients (Figure S3A). However, Treg cells did not prevent the enrichment of the expanded CD4^+^ T cells in TCR Vα2^+^ clones (Figure S3B), suggestive of preferential expansion of FV-specific clones. More importantly, addition of Treg cells did not prevent the migration, accumulation, or local expansion of CD4^+^ T cells (Figure S3C) or the preferential expansion of FV-specific TCR Vα2^+^ clones in the bone marrow (Figure S3D). These results indicated that Treg cells acted on a subsequent step of pathology induction by CD4^+^ T cells, such as effector cytokine production. IFN-γ was the major cytokine produced by the transferred CD4^+^ T cells (Figures 4A–4D), and because this cytokine has an established role in myelosuppression (Raefsky et al., 1985), we examined its role in this model. The additional Treg cells caused, on average, 20% reduction in the percentage of splenic CD4^+^ T cells able to produced IFN-γ upon in vitro restimulation (Figures 4A and 4B). The effect of Treg cells on elicited IFN-γ production by bone marrow CD4^+^ T cells was more dramatic, in which they caused 45% reduction in the percentage of IFN-γ producing cells (Figures 4C and 4D). Furthermore, Treg cells had a profound effect on the amount of IFN-γ produced by individual bone marrow CD4^+^ T cells, on the basis of the median fluorescent intensity (MFI) of the IFN-γ staining, which was reduced by 34% (Figure 4C). The effect of Treg cells on CD4^+^ T cell production of IFN-γ was also evident in IFN-γ serum concentration, which was reduced by 40% (Figure 4E). Thus, Treg cells appeared to be limiting CD4^+^ T cell production of IFN-γ, especially in the local bone marrow environment.

To determine a possible pathogenic role for IFN-γ in FV-induced bone marrow pathology, we used as recipients mice defective in IFN-γ receptor signaling. In comparison to FV-infected *Rag1*^−/−^ recipients of EF4.1 TCRβ-transgenic CD4^+^ T cells, anemia development in FV-infected *Rag1*^−/−^*Ifngr1*^−/−^ recipients was absent or markedly delayed, demonstrating an essential role for IFN-γ signaling in this process (Figure 4F). Late onset of anemia in *Rag1*^−/−^*Ifngr1*^−/−^ was accompanied by development of myeloid leukemia specifically in these hosts, thereby necessitating termination of these experiments. Collectively, these results established a mechanism for virus-specific CD4^+^ T cell-induced anemia in FV-infected *Rag1*^−/−^ hosts and its suppression by Treg cells.

### The Virus-Naive TCR Repertoire of Treg Cells Is Devoid of Virus-Reactive Clones

The pathology-inducing response of EF4.1 TCRβ-transgenic CD4^+^ T cells required activation of FV-specific clones by FV infection, given that it did not develop in uninfected recipients. It was therefore of interest to establish whether the suppressive response of EF4.1 TCRβ-transgenic Treg cells also required activation of FV-specific Treg cell clones during infection or whether this effect was mediated by Treg cells of unrelated specificity. We first confirmed usage of the transgenic TCRβ chain in regulatory CD4^+^ T cells in EF4.1 TCRβ-transgenic mice, by measuring the degree of allelic exclusion of endogenous TCRβ chains. Expression of endogenous TCRβ chains was found in ∼22% of Treg cells, which also displayed higher expression of CD44 than the bulk of Treg cells (Figure S4), suggesting that, similarly to memory CD4^+^ T cells, they may be the result of the exposure to diverse environmental antigens (Fisson et al., 2003). Nevertheless, the TCRβ transgenic chain was used by the majority (78%) of Treg cells in EF4.1 TCRβ-transgenic mice, and it was therefore possible that in some of them it conferred env specificity.

**Figure 5:**
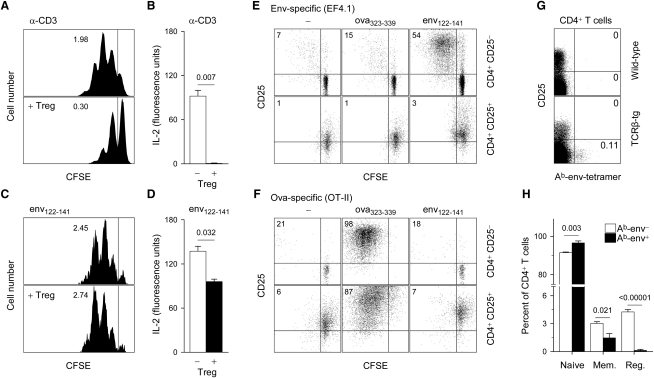
Lack of env Reactivity in EF4.1 TCRβ-Transgenic CD4^+^ Treg Cells (A–D) CFSE dilution profile of conventional EF4.1 TCRβ-transgenic CD4^+^ T cells (A and C) and IL-2 concentration in supernatants (B and D) of cultures of CD45.1^+^ naive CD45RB^hi^CD25^−^ EF4.1 TCRβ-transgenic CD4^+^ T cells, with (+ Treg) or without an equal number of CD45.2^+^ CD25^+^ EF4.1 TCRβ-transgenic Treg cells, stimulated either with anti-CD3 (A and B) or with the env_122-141_ peptide (C and D) presented by bone marrow-derived dendritic cells. IL-2 concentration and CFSE profiles were assayed on days 2 and 3 of culture, respectively. Specifically for env_122–141_ peptide stimulation, the CFSE profile of responding cells only is shown. No differences in the fraction of responding cells were observed in the presence or absence of Treg cells. Numbers within the plots denote the mean division number of responding cells. (E and F) CFSE dilution profile of purified CD25^−^ or CD25^+^ EF4.1 TCRβ-transgenic (E) or OT-II TCRαβ-transgenic (F) CD4^+^ T cells, 5 days after stimulation by bone marrow-derived dendritic cells in the absence of specific peptides (−) or presenting either env_122–141_ or ova_323–339_ peptides. Numbers within the quadrants represent the percentage of divided cells. One experiment, representative of four similar experiments, is shown. (G) A^b^-env tetramer staining and CD25 expression in gated CD4^+^ T cells from wild-type and EF4.1 TCRβ-transgenic mice. Numbers within the quadrants indicate the percentage of positive cells. (H) Percentage of naive, memory (mem.), and regulatory (reg.) cells in gated A^b^-env^−^ or A^b^-env^+^ EF4.1 TCRβ-transgenic CD4^+^ T cells. Numbers within the graph denote the p values. Data were obtained from four mice per group.

To test whether Treg cells in EF4.1 TCRβ-transgenic mice contained any env-specific clones, we examined their potential to suppress a responding CD4^+^ T cell population in an env-specific manner in vitro. CD3-stimulated EF4.1 TCRβ-transgenic Treg cells were able to efficiently suppress the proliferation (Figure 5A) and completely inhibited IL-2 production (Figure 5B) by CD3-stimulated EF4.1 TCRβ-transgenic naive CD4^+^ T cells, demonstrating potent suppressive function by EF4.1 TCRβ-transgenic Treg cells. In contrast, env_122–141_-stimulated EF4.1 TCRβ-transgenic Treg cells had no effect on the proliferation (Figure 5C) and only a small effect on IL-2 production (Figure 5D) by env_122–141_-stimulated EF4.1 TCRβ-transgenic naive CD4^+^ T cells. The small apparent effect of Treg cells on IL-2 production by env-specific naive CD4^+^ T cells was probably due to IL-2 consumption resulting from the high ratio of total Treg cells (50% of culture T cells) to env-specific T cells (2% of culture T cells) in these conditions. Together, these findings suggested lack of specific induction of suppressive function in EF4.1 TCRβ-transgenic Treg cells by the env_122–141_ peptide.

To test the env reactivity of EF4.1 TCRβ-transgenic Treg cells more directly, we measured their proliferative response to env_122–141_ presented by dendritic cells, under conditions previously shown to expand antigen-specific Treg cells (Yamazaki et al., 2003). Treg cell-depleted CD25^−^CD4^+^ EF4.1 TCRβ-transgenic T cells responded specifically to env_122–141_ stimulation by proliferating far more extensively than the spontaneous proliferation seen upon Treg cell removal in the absence of peptide or in the presence of the unrelated ova_323–339_ peptide (Figure 5E). In contrast, no proliferation of EF4.1 TCRβ-transgenic Treg cells was seen after stimulation with either peptide (Figure 5E). As a control for the efficiency of in vitro stimulation of Treg cells, we used CD25^−^CD4^+^ T cells and Treg cells from ovalbumin (ova)-specific OT-II TCR-transgenic mice, which carry TCRα and TCRβ transgenes. Notably, the combination of transgenic TCRα and TCRβ expression was found in almost all naive CD4^+^ T cells, but only 22% of Treg cells from OT-II TCR-transgenic mice (Figure S5), indicating that reactivity to this foreign antigen (ova) was underrepresented in the Treg cell TCR repertoire even in TCRα and TCRβ doubly transgenic mice. However, because TCRα was not subject to allelic exclusion, the relatively small population of Treg cells expressing the OT-II TCRβ transgene was ova specific and proliferated to ova_323–339_, but not env_122–141_ stimulation under these experimental conditions (Figure 5F). Thus, the EF4.1 TCRβ-transgenic Treg cell TCR repertoire appeared to lack clones able to respond to in vitro stimulation with the env_122–141_ peptide, and such a finding is in line with observations that the TCR repertoire of natural Treg cells is biased toward recognition of self-peptides (Hsieh et al., 2004).

To directly visualize env-specific clones in polyclonal EF4.1 TCRβ-transgenic CD4^+^ T cells, we stained these cells with MHC class II A^b^-env_122–141_ tetramers (A^b^-env). Although binding to this reagent is specific to env-reactive clones (MacLeod et al., 2006), binding is also a function of TCR affinity (Crawford et al., 1998) and A^b^-env tetramer binding is restricted only to env-specific clones with a relatively high functional avidity. Indeed, a small fraction of TCRβ-transgenic, but not wild-type control, CD4^+^ T cells were A^b^-env^+^ (Figure 5G). Importantly, A^b^-env^+^ clones were found exclusively in the conventional CD25^−^CD4^+^ T cell subset (Figure 5G). Compared with the bulk A^b^-env^−^ CD4^+^ T cell population, A^b^-env^+^ CD4^+^ T cells were enriched in naive T cells but devoid of Treg cells (Figure 5H). These results showed that env-specific clones with TCR affinity high enough for A^b^-env binding were absent from the TCR repertoire of EF4.1 TCRβ-transgenic Treg cells.

### Suppression of Bone Marrow Pathology by env-Nonspecific Treg Cells

**Figure 6:**
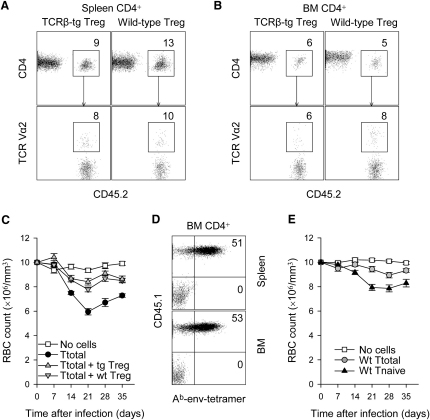
Suppression of env-Specific CD4^+^ T Cell-Induced Bone Marrow Pathology by env-Nonspecific Treg Cells (A and B) Percentage of CD45.2^+^ CD4^+^ Treg cells in total CD4^+^ T cells (top row) and percentage of TCR Vα2^+^ T cells in CD45.2^+^ CD4^+^ Treg cells (bottom row), in the spleen (A) or the bone marrow (B) of FV-infected *Rag1*^−/−^ recipients, after cotransfer of CD45.1^+^ EF4.1 TCRβ-transgenic total CD4^+^ T cells with either EF4.1 TCRβ-transgenic (TCRβ-tg) or wild-type CD45.2^+^ CD25^+^ CD4^+^ Treg cells, at 1:1 ratio. Numbers within the plots indicate the percentage of positive cells and are representative of six to eight mice per group, analyzed on day 21 after transfer. (C) Changes in RBC counts in FV-infected *Rag1*^−/−^ mice, which received total EF4.1 TCRβ-transgenic CD4^+^ T cells, alone (Ttotal) or together with either EF4.1 TCRβ-transgenic (Ttotal + tg Treg) or wild-type (Ttotal + wt Treg) CD25^+^ CD4^+^ Treg cells. Values represent the mean (±SEM) of six to ten mice per group per time point analyzed in three independent experiments. p < 0.0003 between “Ttotal” and either “Ttotal + tg Treg” or “Ttotal + wt Treg” on day 21. (D) A^b^-env tetramer staining in CD4^+^ T cells from the spleen or the bone marrow of FV-infected *Rag1*^−/−^ recipients, after cotransfer of CD45.1^+^ EF4.1 TCRβ-transgenic total CD4^+^ T cells with CD45.2^+^ wild-type CD25^+^ CD4^+^ Treg cells, at 1:1 ratio. Numbers within the quadrants indicate the percentage of tetramer positive cells in either CD45.1^+^ or CD45.2^+^ CD4^+^ T cells and are representative of four mice per group, analyzed on day 21 after transfer. (E) Changes in RBC counts in FV-infected *Rag1*^−/−^ mice, which received either total CD4^+^ T cells (wt Ttotal) or purified naive CD45RB^hi^CD25^−^ CD4^+^ T cells (wt Tnaive) from wild-type donor mice or no T cells (no cells). Values represent the mean (±SEM) of nine to ten mice per group per time point analyzed in two independent experiments. p < 0.0003 between “wt Tnaive” and “wt Ttotal,” on day 21.

Virus-naive EF4.1 TCRβ-transgenic Treg cells appeared to lack env-specific clones, yet they efficiently suppressed the development of bone marrow pathology induced by FV-infection. We therefore addressed whether suppression of FV-induced pathology was mediated by in vivo expansion of a small number of FV-specific EF4.1 TCRβ-transgenic Treg cells, the initial frequency of which may have been too low to measure with the methods we employed. EF4.1 TCRβ-transgenic Treg cells were compared with nontransgenic control Treg cells, in which env-specific clones would be at a much lower frequency or absent. Expansion of CD45.2^+^ EF4.1 TCRβ-transgenic and wild-type Treg cells was comparable, and both reached similar proportions of total CD4^+^ T cells in cotransfers with pathogenic CD45.1^+^ EF4.1 TCRβ-transgenic CD4^+^ T cells, in the spleen (Figure 6A) and the bone marrow (Figure 6B) of FV-infected recipients. Furthermore, in contrast to pathogenic EF4.1 TCRβ-transgenic CD4^+^ T cells (Figure S3), neither EF4.1 TCRβ-transgenic nor wild-type Treg cells showed any enrichment for TCR Vα2^+^ clones (Figures 6A and 6B), indicating that their expansion was polyclonal and not skewed by expansion of env-specific clones. More importantly, wild-type Treg cells were able to suppress induction of anemia by pathogenic FV-specific CD4^+^ T cells, with efficiency similar to EF4.1 TCRβ-transgenic Treg cells (Figure 6C). Suppression of bone marrow pathology by wild-type Treg cells was not the result of selective expansion of env-specific clones because, in contrast to pathogenic EF4.1 TCRβ-transgenic CD4^+^ T cells, half of which stained brightly with the A^b^-env tetramer, no-A^b^-env^+^ cells were found in expanded wild-type Treg cell population (Figure 6D). Thus, suppression of FV-specific CD4^+^ T cell-induced bone marrow pathology was mediated by Treg cells independently of shared antigenic specificity.

Although suppression of anemia development depended on the cotransfer of env-nonspecific Treg cells, it was possible that env-specific Treg cells were generated de novo from EF4.1 TCRβ-transgenic CD4^+^ T cells during infection and contributed to disease suppression. Furthermore, although Treg cells completely lacked env specificity, they may have reacted to other FV-encoded epitopes. For evaluation of induction of FoxP3 expression in FV-specific CD4^+^ T cells responding to infection, EF4.1 TCRβ-transgenic mice were crossed to a FoxP3EGFP reporter strain, *Foxp3^egfp^* (Wang et al., 2008) (Figure S6A). Despite marked expansion in response to FV infection (Figure S6B), no FoxP3 expression was detected in purified FoxP3^−^ (GFP^−^) CD45.1^+^ EF4.1 TCRβ-transgenic *Foxp3^egfp^* CD4^+^ T cells adoptively transferred into FV-infected B6 recipients (Figure S6C). Similarly, no FoxP3 expression was detected in purified FoxP3^−^ (GFP^−^) CD45.1^+^ EF4.1 TCRβ-transgenic *Foxp3^egfp^* CD4^+^ T cells cotransferred with CD45.2^+^ wild-type Treg cells into FV-infected *Rag1*^−/−^ recipients (Figure S6D). Furthermore, FV infection did not expand any Treg cell subpopulation with potential FV reactivity because no in vitro proliferative response to dendritic cells pulsed with env_122–141_ or whole FV could be seen in Treg cells isolated from the spleens of FV-infected B6 mice (Figure S7A) or the bone marrow of FV-infected *Rag1*^−/−^ recipients of EF4.1 TCRβ-transgenic CD4^+^ T cells and wild-type Treg cells (Figure S7B). Therefore, FV infection did not induce the expansion of pre-existing Treg cells or the conversion into Treg cells of CD4^+^ T cells, which were able to respond to FV-encoded env or other potential epitopes.

Our data indicated that development of anemia in this model was caused by FV-specific CD4^+^ T cells but could be suppressed by any Treg cell population, regardless of FV reactivity. If so, then CD4^+^ T cells from nontransgenic wild-type donors, in which the frequency of FV-specific pathogenic clones would be physiologically very low (<10^−5^) but the frequency of Treg cells is normal (10^−1^), should not induce anemia. However, removal of Treg cells from this population should unmask the pathogenic potential of the relative infrequent FV-specific clones. Indeed, in contrast to total CD4^+^ T cells from EF4.1 TCRβ-transgenic mice (Figure 2A), those from wild-type mice failed to induce severe anemia upon transfer into FV-infected *Rag1*^−/−^ recipients (Figure 6E). Importantly, Treg cell depletion allowed naive wild-type CD4^+^ T cells to induced anemia in FV-infected recipients (Figure 6E), the severity of which was milder than anemia induced by naive EF4.1 TCRβ-transgenic CD4^+^ T cells, in line with the reduced frequency of FV-specific clones in the former population. Thus, development of bone marrow pathology under these experimental conditions crucially depended on the relative frequency of conventional CD4^+^ T cells with FV specificity and Treg cells independently of FV specificity.

### Potential Mechanisms of Treg Cell Activation and Action in FV Infection

**Figure 7:**
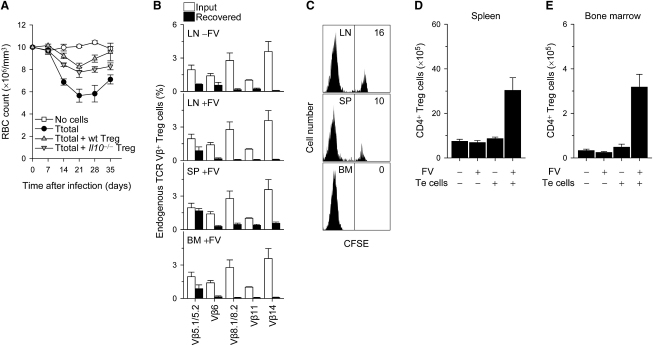
Potential Mechanisms of Treg Cell Activation and Action in FV-Induced Bone Marrow Pathology (A) Changes in RBC counts in FV-infected *Rag1*^−/−^ mice, which received either no T cells (no cells) or total EF4.1 TCRβ-transgenic CD4^+^ T cells either alone (Ttotal) or together with wild-type (Ttotal + wt Treg) or IL-10-deficient CD25^+^ CD4^+^ Treg cells (Ttotal + *Il10^−/−^* Treg). Values represent the mean (±SEM) of five to six mice per group per time point analyzed in a single experiment. p < 0.001 and p < 0.02 between “Ttotal” and “Ttotal + *Il10^−/−^* Treg,” on days 14 and 21, respectively; the p value was not significant between “Ttotal + wt Treg” and “Ttotal + *Il10^−/−^* Treg.” (B) Expression of individual endogenous TCR Vβ5.1/5.2, Vβ6, Vβ8.1/8.2, Vβ11, and Vβ14 chains in EF4.1 TCRβ-transgenic Treg cells recovered from the lymph nodes (LNs), the spleen (SP) or the bone marrow (BM) of uninfected (− FV) or FV-infected (+ FV) *Rag1*^−/−^ recipients, which received CD45.2^+^ EF4.1 TCRβ-transgenic CD25^+^ CD4^+^ Treg cells together with CD45.1^+^ EF4.1 TCRβ-transgenic total CD4^+^ T cells, at 1:1 ratio. Expression in EF4.1 TCRβ-transgenic Treg cells before (input) and 21 days after transfer (recovered) is shown. Values represent the mean (±SEM) of two to four individual recipients from a single experiment. (C) CFSE dilution profile of CD45.1^+^ CD4^+^ Treg cells in the lymph nodes (LN), spleen (SP), and bone marrow (BM) of FV-infected *Rag1*^−/−^ recipients, which received CD45.2^+^ EF4.1 TCRβ-transgenic total CD4^+^ T cells, at the time of FV infection, and a second cohort of CFSE-labeled CD45.1^+^ wild-type CD25^+^ CD4^+^ Treg cells 21 days later. Organs were analyzed 3 days after transfer of the second Treg cell cohort. Numbers within the quadrants denote the percentage of undivided cells and are representative of four mice analyzed in two separate experiments. p < 0.007 between “BM” and either “LN” or “SP.” (D and E) Absolute number of CD45.2^+^ Treg cells recovered from the spleen (D) or the bone marrow (E) of uninfected (− FV) or FV-infected (+ FV) *Rag1*^−/−^ recipients, which received CD45.2^+^ wild-type CD25^+^ CD4^+^ Treg cells alone (− Te cells) or together with CD45.1^+^ EF4.1 TCRβ-transgenic total CD4^+^ T cells at 1:1 ratio (+ Te cells). Values represent the mean (±SEM) of three to six mice per group analyzed on day 21.

Nonshared antigen specificity between pathogenic CD4^+^ T cells and Treg cells suggested a broad mode of Treg cell-mediated suppression. Although the precise mechanisms underlying Treg cell-mediated protection against bone marrow pathology are currently unclear, a contribution of Treg cell-derived IL-10 was excluded, given that anemia development was comparably suppressed by either wild-type or IL-10-deficient Treg cells (Figure 7A). Lack of FV specificity in Treg cells suppressing anemia induction by pathogenic FV-specific CD4^+^ T cells also raised the important question of whether or how Treg cells were being activated to mediate this suppression. Transfer of Treg cells into *Rag1*^−/−^ recipients was associated with elevated expression of ICOS and CD103, which was not, however, further enhanced by FV infection (not shown). Similarly, transfer of EF4.1 TCRβ-transgenic Treg cells into *Rag1*^−/−^ recipients led to enrichment of clones expressing the transgenic, rather than endogenous, TCRβ chains (Figure 7B). However, the pattern of TCRβ expression in EF4.1 TCRβ-transgenic Treg cells was not affected by FV infection or the location, from which Treg cells were recovered (Figure 7B), suggesting that Treg cell recruitment and/or expansion in the infected bone marrow was independent of TCR specificity.

Because transfer of Treg cells into lymphopenic *Rag1*^−/−^ recipients had the potential to cause their activation irrespective of FV-infection, we next examined whether Treg cells were receiving additional signals originating from the infection itself. To assess their proliferative response during the course of FV infection, we adoptively transferred a cohort of CFSE-labeled sensor Treg cells into *Rag1*^−/−^ recipients 21 days after FV infection and primary transfer of EF4.1 TCRβ-transgenic CD4^+^ T cells.

At this time point after infection, T cell numbers in lymphoid organs of *Rag1*^−/−^ recipients were equivalent to those in T cell-replete wild-type mice, because of significant expansion of the first cohort of EF4.1 TCRβ-transgenic CD4^+^ T cells (Figure 2I). The second cohort of sensor Treg cells displayed extensive proliferation in the lymph nodes, spleen, and bone marrow of recipient mice 3 days after transfer, with substantially fewer undivided cells in the bone marrow than in the spleen or lymph nodes (Figure 7C). Although this analysis did not discriminate between enhanced division in the bone marrow or preferential migration of divided cells into the bone marrow, it revealed that Treg cell activation to enter cell division continued throughout the course of FV infection, despite the presence of substantial numbers of T cells, and that Treg cells recovered from the bone marrow were associated with higher proliferative history. To compare the degree of Treg cell activation directly by the infection, the presence of pathogenic CD4^+^ T cells, or the transfer into lymphopenic *Rag1*^−/−^ recipients, we further examined their individual or combined effect on Treg cell expansion. Recovery of Treg cells from recipient mice was similar when Treg cells were transferred into uninfected *Rag1*^−/−^ recipients either alone or together with EF4.1 TCRβ-transgenic CD4^+^ T cells or alone into FV-infected *Rag1*^−/−^ recipients (Figures 7D and 7E). In contrast, recovery of Treg cells was three and six times greater in the spleen and bone marrow, respectively, when Treg cells were transferred together with EF4.1 TCRβ-transgenic CD4^+^ T cells into FV-infected *Rag1*^−/−^ recipients, than in any other combination (Figures 7D and 7E). These results argued against a direct response of Treg cells to FV infection or transfer into *Rag1*^−/−^ recipients and instead indicated that Treg cell expansion and/or survival depended on the presence of CD4^+^ T cells responding to FV infection.

## Discussion

Strong and lasting antiviral T cell responses, which do not have an impact on virus replication, have the potential to cause immune pathology. Our results demonstrated that the local IFN-γ production by virus-specific CD4^+^ T cells in response to unresolving FV infection led to bone marrow pathology, which manifested as anemia. Importantly, the severity of FV-induced CD4^+^ T cell-mediated anemia critically depended on the balance between FV-specific pathogenic CD4^+^ T cells and Treg cells. Moreover, efficient suppression of FV-induced bone marrow pathology by Treg cells did not necessitate direct viral-antigen recognition by Treg cells because the TCR repertoire of Treg cells in virus-naive and FV-infected mice was lacking virus-specific clones and no conversion of FV-specific pathogenic CD4^+^ T cells into FoxP3-expressing Treg cells occurred during infection.

Bone marrow pathology is a common feature of retroviral infection in humans (Moses et al., 1998; Kulkosky et al., 2000; Redd et al., 2007; Mlisana et al., 2008). Bone marrow dysfunction is responsible for anemia development in approximately 60% and 90% of acute and chronic HIV-1 infection, respectively, and is a substantial contributor to other cytopenias, including reduced lymphocyte production (Moses et al., 1998; Kulkosky et al., 2000; Redd et al., 2007; Mlisana et al., 2008). It is currently unclear whether bone marrow pathology in HIV-1 infection results from direct cytopathic effects of the virus on bone marrow precursors or from indirect immune-mediated effects, and it is likely that its origin is multifactorial (Molina et al., 1990; Moses et al., 1998; Kulkosky et al., 2000; Redd et al., 2007; Mlisana et al., 2008). Clades of HIV-1, which differ in their ability to infect bone marrow precursors, also differ in their ability to induce severe anemia (Redd et al., 2007; Mlisana et al., 2008). Nevertheless, anemia in HIV-1 infection is also observed in the absence of bone marrow-precursor infection, indicating an indirect mechanism of bone marrow dysfunction (Molina et al., 1990; Moses et al., 1998; Kulkosky et al., 2000). Studies in mice with unresolving noncytopathic LCMV infection due to perforin deficiency have demonstrated that excessive virus-specific CD8^+^ T cell, but not CD4^+^ T cell, response results in bone marrow pathology, which is, in part, IFN-γ mediated (Binder et al., 1998). Moreover, aplastic anemia can be one of the consequences of chronic graft-versus-host (GvH) reaction driven by histocompatibility disparities, in the absence of infection (Sprent et al., 1994; Chen et al., 2004). Alloantigen-specific CD4^+^ T cells can contribute to GvH-driven aplastic anemia either directly, by recognition of major histocompatibility alloantigens on bone marrow precursors (Sprent et al., 1994), or indirectly, by provision of help to minor histocompatibility alloantigen-specific CD8^+^ T cells (Chen et al., 2004). Similarly, we found that FV infected a large number of bone marrow precursors with no apparent consequences for bone marrow function in the absence of an adaptive virus-specific immune response. Excessive virus-specific CD4^+^ T cell response was then directly responsible for development of bone marrow pathology in an IFN-γR-dependent manner. Collectively, these findings emphasize the harmful contribution of indirect mechanisms of T cell-mediated immune pathology underlying the development of anemia in chronic viral infection.

In contrast to pathology induced by direct cytopathic effects of viral infection, T cell-mediated immune pathology is amenable to immune regulation. Thus, Treg cells in the transferred CD4^+^ T cell population from wild-type donor mice, in which the precursor frequency of FV-specific CD4^+^ T cells is physiologically very low, mediated almost complete protection from the development of bone marrow pathology. However, Treg cell removal from this population unmasked the pathogenic potential of FV-specific CD4^+^ T cells. This balance of Treg cells and FV-specific CD4^+^ T cells was markedly affected in EF4.1 TCRβ-transgenic CD4^+^ T cells, as a result of the increase in the frequency of FV-specific conventional CD4^+^ T cell clones; such an increase could cause bone marrow pathology despite the presence of Treg cells. Nevertheless, Treg cell removal from the EF4.1 TCRβ-transgenic CD4^+^ T cell population also exacerbated bone marrow pathology, indicating that Treg cells contained in this population mediated substantial, though incomplete, protection. Furthermore, enrichment of the EF4.1 TCRβ-transgenic CD4^+^ T cell population with additional Treg cells was protective against bone marrow pathology by inhibiting the local IFN-γ production by pathogenic CD4^+^ T cells, which would otherwise adversely affect bone marrow function. Therefore, our results demonstrated that, at least in unresolving FV infection, CD4^+^ T cell-mediated bone marrow pathology was triggered by Treg cell insufficiency and suggested that similar mechanisms may operate in HIV-1 infection. Although the precise fate of Treg cells in HIV-1 infection is currently controversial, Treg cells are susceptible to HIV-1 infection and a reduction in Treg cell numbers and function has been correlated by certain studies with immune activation (Kinter et al., 2004; Eggena et al., 2005). Moreover, relative Treg cell deficiency is found in almost all patients with acquired aplastic anemia of suspected autoimmune etiology (Solomou et al., 2007), and adoptive transfer of Treg cells in a mouse model can ameliorate aplastic anemia driven by minor histocompatibility differences (Chen et al., 2007). Thus, Treg cells may play an important role in protecting the bone marrow against a diverse spectrum of immune pathologies.

Despite the fundamental importance of Treg cells for immune regulation, our knowledge of their antigenic specificity remains incomplete. The majority of experimental evidence suggests that Treg cells carry a distinct TCR repertoire, which is biased toward recognition of self-peptide-MHC II complexes with higher-than-average avidity (Hsieh et al., 2004; Sakaguchi, 2004; Fontenot and Rudensky, 2005; Picca et al., 2006). However, a certain degree of self-reactivity is a property of all T cells, because it represents a requirement for positive selection, and is not predictive of lack of reactivity to foreign peptides. Our results with mice bearing a polyclonal TCR repertoire with reduced diversity due to expression of a TCRβ transgene, an approach previously shown to be compatible with Treg cell development (Hsieh et al., 2004; Pacholczyk et al., 2006), demonstrated that clones with reactivity to FV env_122–141_ were strikingly underrepresented in the Treg cell TCR repertoire of virus-naive mice. It has been recently shown that clones reactive to a variant of the MHC class II Eα_52–68_ peptide, which is a foreign peptide for B6 mice, are similarly underrepresented in the Treg cell pool (Burchill et al., 2008). Thus, it is conceivable that the TCR repertoire of naturally occurring Treg cells is not normally equipped to detect foreign peptides.

Although the emergence of pathogen-specific suppressive T cells has been suggested by studies of chronic infection, it has rarely been clear whether pathogen-specific suppression is mediated by naturally occurring Treg cells or by one of many different types of T cells with inducible suppressive activity (Mills, 2004; Rouse et al., 2006; Belkaid, 2007). A population of T cells at the site of chronic *Leishmania major* infection, originating from naturally occurring Treg cells, has been shown to react to *L. major*-infected dendritic cells (Suffia et al., 2006). Although the precise nature of *L. major* antigens recognized by Treg cells in this case is not known, this study emphasizes that pathogen-specific Treg cells can be found. However, *L. major* contains 8300 protein-coding genes and it is perhaps unsurprising that the Treg cell TCR repertoire did not completely lack reactivity to products of the entire *L. major* 32.8Mb genome. In contrast, clones specific to FV, the 8.3 Kb genome of which generates only three polypeptides containing one described MHC class II A^b^-restricted epitope (Iwashiro et al., 1993), may not necessarily develop. Indeed, although Treg cell reactivity to env_122–141_ was assessed more rigorously than reactivity to other potential viral epitopes, our results strongly suggested that FV-specific clones were not present in the virus-naive Treg cell TCR repertoire, nor did they arise from expansion of preexisting Treg cells or conversion of FV-specific conventional CD4^+^ T cells into Treg cells in response to FV infection. Nevertheless, our results also indicated that reactivity to FV-derived antigens did not represent an absolute requirement for regulation of virus-induced immune pathology. Although the effects of lymphopenia in adoptive hosts could not be excluded, our results showed that the most important factor affecting expansion of Treg cell numbers during FV infection was, in fact, the concomitant response to FV of a conventional effector CD4^+^ T cell population, which could be providing an essential Treg cell growth factor, such as IL-2 (Almeida et al., 2006) or inducing elevated expression of Treg cell-stimulating self-peptide-MHC II complexes. Thus, strategies for enhancement of Treg cell numbers or function, irrespective of peptide specificity, may prove beneficial in the prevention and treatment of bone marrow immune pathology.

## Experimental Procedures

### Mice

Inbred C57BL/6 (B6) and CD45.1-congenic B6 mice (B6.SJL-*Ptprc^a^ Pep3^b^*/BoyJ) were originally obtained from the Jackson Laboratory (Bar Harbor, ME, USA) and were subsequently maintained at NIMR animal facilities. B6-backcrossed Rag1-deficient mice (Mombaerts et al., 1992) (B6.129S7-*Rag1^tm1Mom^*/J or *Rag1^−/−^*), IFN-γR1-deficient mice (Huang et al., 1993) (B6.129S7-*Ifngr1^tm1Agt^*/J or *Ifngr1^−/−^*), B cell-deficient mice (Kitamura et al., 1991) (B6.129S2-*Igh-6^tm1Cgn^*/J or *Igh6^−/−^*, also known as μMT), IL-10-deficient mice (Kuhn et al., 1993) (B6.129P2-*Il10^tm1Cgn^*/JLt or *Il10^−/−^*), FoxP3EGFP reporter mice (B6-*Foxp3^tm1Mal^* or *Foxp3^egfp^*) (Wang et al., 2008), and A^b^-ova_323-339_-specific OT-II TCR-transgenic mice (Barnden et al., 1998) have been previously described and were also maintained at NIMR animal facilities. F-MuLV env-specific TCR-transgenic mice were generated by pronuclear microinjection of genes encoding the TCRα and TCRβ chains of an env-specific CD4^+^ T cell clone into fertilized B6 oocytes. In brief, we fused the SB14-31 T cell clone (Iwashiro et al., 1993), specific to env_122–141_ presented by MHC II A^b^, to TCRαβ-negative BW5147 thymoma cells to produce the 1A6 hybridoma cell line, which was then used as source for RNA. TCRα and TCRβ cDNAs were cloned and inserted into the hCD2-VA expression cassette (Zhumabekov et al., 1995), which directs expression in T cells. Specifically for the TCRβ chain, a cDNA-genomic DNA hybrid was used, in which intron 1 of the TCRVβ-encoding gene (*Tcrbv1s1*) was preserved. For the present study, a TCR-transgenic line (EF4.1) expressing only the TCRβ chain of the SB14-31 clone was selected, to maintain a polyclonal TCR repertoire. Lack of transgenic TCRα chain expression was confirmed by crossing to B6-*Rag1*^−/−^ and B6-*Tcra*^−/−^ mice (Philpott et al., 1992). All animal experiments were conducted according to UK Home Office regulations and local guidelines.

### Friend Virus Infection

The Friend virus (FV) used in this study was a retroviral complex of a replication-competent B-tropic helper murine leukemia virus (F-MuLV) and a replication-defective polycythemia-inducing spleen focus-forming virus (SFFVp). FV was propagated in vivo and prepared as 10% w/v homogenate from the spleen of 12-day-infected BALB/c mice and was free of lactate dehydrogenase-elevating virus (LDV). *Rag1^−/−^* mice received an inoculum of ∼1000 spleen focus-forming units (SFFUs) of FV, injected via the tail vein in 0.1 ml of phosphate-buffered saline. B6 and *Igh6^−/−^* mice received a higher dose of FV containing ∼10,000 SFFUs. We estimated cell-associated virus in infected mice by flow-cytometric detection of infected cells using surface staining for the glycosylated product of the viral *gag* gene (glyco-Gag), using the matrix (MA)-specific monoclonal antibody 34 (mouse IgG2b), and subsequent use of an anti-mouse IgG2b-FITC secondary reagent (BD Biosciences, San Jose, CA, USA).

### Assessment of Primary and Secondary Lymphoid Organ Cellularity

Bone marrow cells were isolated by flushing the bone cavities of tibiae and femurs and bone marrow cellularity was assessed based on the cellular contents of these locations. Single-cell suspensions were prepared from the spleen and lymph nodes of mice following mechanical disruption of the organs on nylon mesh. Spleen suspensions were treated with ammonium chloride for erythrocyte lysis. Lymph node cellularity was calculated as the sum of the cellular contents of inguinal, axillary, brachial, mesenteric and superficial cervical lymph nodes.

### Assessment of Anemia

Mice were bled by a small incision of the tail vein and blood was collected into heparinized capillary tubes. Complete blood counts were measured on a VetScan HMII hematology analyzer (Abaxis, CA, USA), in accordance with the manufacturer's instructions.

### T Cell Purification and Adoptive Transfer

CD4^+^ or CD25^+^ T cells were isolated from the spleen and lymph nodes of donor mice, with immunomagnetic positive selection (EasySep beads, StemCell Technologies, Vancouver, BC, Canada) according to the manufacturer's instructions. Enriched cell suspensions were stained with antibodies to surface markers and then further purified by cell sorting, performed on MoFlo cell sorters (Dako, Fort Collins, CO, USA). Typical cell purity after cell sorting was higher than 98%. Purified cells (1 × 10^6^ per recipient) were injected in recipient mice via the tail vein in 0.1 ml of air-buffered IMDM.

### Flow-Cytometric Analysis

Cells were stained with directly conjugated antibodies to surface markers, obtained from eBiosciences (San Diego, CA, USA), CALTAG/Invitrogen (Carlsbad, CA, USA), or BD Biosciences. Four- and eight-color cytometry were performed on FACSCalibur (BD Biosciences) and CyAn (Dako) flow cytometers, respectively, and analyzed with FlowJo v8.7 (Tree Star, Ashland, OR, USA) or Summit v4.3 (Dako) analysis software, respectively. For detection of cytokine synthesis, cells were stained for surface markers and stimulated for 4 hr with PdBu and ionomycin (both at 500 ng/ml), in the presence of Monensin (1 μg/ml). Cells were then fixed and permeabilized with buffers from eBiosciences, before intracellular staining with IL-17A and IFN-γ-specific antibodies (eBiosciences). FoxP3 was detected by intranuclear staining with a FoxP3-staining kit (eBiosciences), according to the manufacturer's instructions. A^b^-env_122-141_ tetramers (kindly provided by D. Gray, University of Edinburgh, UK) were prepared and used as previously described (Crawford et al., 1998; MacLeod et al., 2006). In brief, cells were incubated with PE-labeled A^b^-env_122-141_ tetramers at 37°C in IMDM containing 5% FCS; this was followed by staining with antibodies to surface markers, before analysis.

### Analysis of Serum Cytokine Concentrations

Serum was prepared from blood samples, which were allowed to clot at 4°C. Serum cytokine concentrations were analyzed by a Bio-plex cytokine assay (Bio-Rad Laboratories Ltd. UK) on a Luminex 100 instrument (Bio-Rad Laboratories Ltd. UK) according to manufacturer's instruction.

### In Vitro T Cell Activation and Suppression

Single-cell suspensions were prepared from the spleen or lymph nodes of donor mice and 0.5 × 10^6^ cells per well were stimulated in 96-well plates with the indicated amount of various stimuli. Alternatively, 0.5 × 10^6^ naive CD45RB^hi^CD25^−^CD4^+^ T cells or CD25^+^CD4^+^ Treg cells, purified by flow-cytometric sorting, were stimulated separately or mixed together at 1:1 ratio by added APCs. B cells, macrophages, or dentritric cells were used as APCs with comparable results. B cells were purified with immunomagnetic-positive selection (EasySep beads, StemCell Technologies) from the spleen of donor B6 or *Tcra*^−/−^ mice. Macrophages were isolated from peritoneal cavity exudate cells after adherence for 1 hr to plastic plates containing tissue culture. Dendritic cells were prepared from GM-CFS-treated bone marrow cultures. Cultures were stimulated with either anti-CD3 antibodies (at 0.5 μg/ml) or with titrated amounts of env_122–141_ (DEPLTSLTPRCNTAWNRLKL) or ova_323–339_ (ISQAVHAAHAEINEAGR) peptides. Specifically for the activation of CD25^+^CD4^+^ Treg cells (Figures 5E and 5F), cultures were stimulated in the absence or in the presence of 20 U/ml recombinant IL-2, with similar results. T cell activation was assessed 18 hr later by flow-cytometric detection of CD69 or CD154 (CD40L) upregulation (both antibodies from eBiosciences), with comparable results. For CD154 detection, the anti-CD154 antibody was added at the beginning of the culture. IL-2 was detected in culture supernatants collected at 48 hr, with an AlamarBlue (Invitrogen)-based CTLL-2 assay. For assessment of T cell activation on day 3, cells were labeled with CFSE before stimulation and responding cells were identified by CFSE dilution.

### Statistical Analysis

Statistics were generated by Student's t test performed with SigmaPlot v10 software (Systat Software, San Jose, CA, USA).

## Supplemental Data

Document S1. Supplemental Experimental Procedures and Seven Figures

## References

[bib1] Almeida A.R.M., Zaragoza B., Freitas A.A. (2006). Indexation as a novel mechanism of lymphocyte homeostasis: The number of CD4+CD25+ regulatory T cells is indexed to the number of IL-2-producing cells. J. Immunol..

[bib2] Barnden M.J., Allison J., Heath W.R., Carbone F.R. (1998). Defective TCR expression in transgenic mice constructed using cDNA-based alpha- and beta-chain genes under the control of heterologous regulatory elements. Immunol. Cell Biol..

[bib3] Belkaid Y. (2007). Regulatory T cells and infection: A dangerous necessity. Nat. Rev. Immunol..

[bib4] Binder D., van den Broek M.F., Kagi D., Bluethmann H., Fehr J., Hengartner H., Zinkernagel R.M. (1998). Aplastic anemia rescued by exhaustion of cytokine-secreting CD8+ T Cells in persistent infection with lymphocytic choriomeningitis virus. J. Exp. Med..

[bib5] Burchill M.A., Yang J., Vang K.B., Moon J.J., Chu H.H., Lio C.W., Vegoe A.L., Hsieh C.S., Jenkins M.K., Farrar M.A. (2008). Linked T cell receptor and cytokine signaling govern the development of the regulatory T cell repertoire. Immunity.

[bib6] Chen J., Ellison F.M., Eckhaus M.A., Smith A.L., Keyvanfar K., Calado R.T., Young N.S. (2007). Minor antigen H60-mediated aplastic anemia is ameliorated by immunosuppression and the infusion of regulatory T cells. J. Immunol..

[bib7] Chen J., Lipovsky K., Ellison F.M., Calado R.T., Young N.S. (2004). Bystander destruction of hematopoietic progenitor and stem cells in a mouse model of infusion-induced bone marrow failure. Blood.

[bib8] Crawford F., Kozono H., White J., Marrack P., Kappler J. (1998). Detection of antigen-Specific T cells with multivalent soluble class II MHC covalent peptide complexes. Immunity.

[bib9] Eggena M.P., Barugahare B., Jones N., Okello M., Mutalya S., Kityo C., Mugyenyi P., Cao H. (2005). Depletion of regulatory T cells in HIV infection is associated with immune activation. J. Immunol..

[bib10] Fisson S., Darrasse-Jeze G., Litvinova E., Septier F., Klatzmann D., Liblau R., Salomon B.L. (2003). Continuous activation of autoreactive CD4+ CD25+ regulatory T cells in the steady state. J. Exp. Med..

[bib11] Fontenot J.D., Rudensky A.Y. (2005). A well adapted regulatory contrivance: Regulatory T cell development and the forkhead family transcription factor Foxp3. Nat. Immunol..

[bib12] Guidotti L.G., Chisari F.V. (2006). Immunobiology and pathogenesis of viral hepatitis. Annu Rev Pathol..

[bib13] Hasenkrug K.J., Chesebro B. (1997). Immunity to retroviral infection: The Friend virus model. Proc. Natl. Acad. Sci. USA.

[bib14] Hsieh C.S., Liang Y., Tyznik A.J., Self S.G., Liggitt D., Rudensky A.Y. (2004). Recognition of the peripheral self by naturally arising CD25+ CD4+ T cell receptors. Immunity.

[bib15] Huang S., Hendriks W., Althage A., Hemmi S., Bluethmann H., Kamijo R., Vilcek J., Zinkernagel R.M., Aguet M. (1993). Immune response in mice that lack the interferon-gamma receptor. Science.

[bib16] Iwashiro M., Kondo T., Shimizu T., Yamagishi H., Takahashi K., Matsubayashi Y., Masuda T., Otaka A., Fujii N., Ishimoto A. (1993). Multiplicity of virus-encoded helper T-cell epitopes expressed on FBL-3 tumor cells. J. Virol..

[bib17] Iwashiro M., Peterson K., Messer R.J., Stromnes I.M., Hasenkrug K.J. (2001). CD4+ T cells and gamma interferon in the long-term control of persistent Friend retrovirus infection. J. Virol..

[bib18] Kinter A.L., Hennessey M., Bell A., Kern S., Lin Y., Daucher M., Planta M., McGlaughlin M., Jackson R., Ziegler S.F., Fauci A.S. (2004). CD25+CD4+ regulatory T cells from the peripheral blood of asymptomatic HIV-infected individuals regulate CD4+ and CD8+ HIV-specific T Cell immune responses in vitro and are associated with favorable clinical markers of disease status. J. Exp. Med..

[bib19] Kitamura D., Roes J., Kuhn R., Rajewsky K. (1991). A B cell-deficient mouse by targeted disruption of the membrane exon of the immunoglobulin mu chain gene. Nature.

[bib20] Kronenberg M., Rudensky A. (2005). Regulation of immunity by self-reactive T cells. Nature.

[bib21] Kuhn R., Lohler J., Rennick D., Rajewsky K., Muller W. (1993). Interleukin-10-deficient mice develop chronic enterocolitis. Cell.

[bib22] Kulkosky J., Bouhamdan M., Geist A., Nunnari G., Phinney D.G., Pomerantz R.J. (2000). Pathogenesis of HIV-1 infection within bone marrow cells. Leuk. Lymphoma.

[bib23] MacLeod M., Kwakkenbos M.J., Crawford A., Brown S., Stockinger B., Schepers K., Schumacher T., Gray D. (2006). CD4 memory T cells survive and proliferate but fail to differentiate in the absence of CD40. J. Exp. Med..

[bib24] Mills K.H.G. (2004). Regulatory T cells: Friend or foe in immunity to infection?. Nat. Rev. Immunol..

[bib25] Mlisana K., Auld S.C., Grobler A., van Loggerenberg F., Williamson C., Iriogbe I., Sobieszczyk M.E., Abdool Karim S.S. (2008). Anaemia in acute HIV-1 subtype C infection. PLoS ONE.

[bib26] Molina J.M., Scadden D.T., Sakaguchi M., Fuller B., Woon A., Groopman J.E. (1990). Lack of evidence for infection of or effect on growth of hematopoietic progenitor cells after in vivo or in vitro exposure to human immunodeficiency virus. Blood.

[bib27] Mombaerts P., Iacomini J., Johnson R.S., Herrup K., Tonegawa S., Papaioannou V.E. (1992). RAG-1-deficient mice have no mature B and T lymphocytes. Cell.

[bib28] Moses A., Nelson J., Bagby G.C. (1998). The influence of human immunodeficiency Virus-1 on hematopoiesis. Blood.

[bib29] Moskophidis D., Lechner F., Pircher H., Zinkernagel R.M. (1993). Virus persistence in acutely infected immunocompetent mice by exhaustion of antiviral cytotoxic effector T cells. Nature.

[bib30] Pacholczyk R., Ignatowicz H., Kraj P., Ignatowicz L. (2006). Origin and T cell receptor diversity of Foxp3+CD4+CD25+ T cells. Immunity.

[bib31] Pacholczyk R., Kern J., Singh N., Iwashima M., Kraj P., Ignatowicz L. (2007). Nonself-antigens are the cognate specificities of Foxp3+ regulatory T cells. Immunity.

[bib32] Philpott K.L., Viney J.L., Kay G., Rastan S., Gardiner E.M., Chae S., Hayday A.C., Owen M.J. (1992). Lymphoid development in mice congenitally lacking T cell receptor alpha beta-expressing cells. Science.

[bib33] Picca C.C., Larkin J., Boesteanu A., Lerman M.A., Rankin A.L., Caton A.J. (2006). Role of TCR specificity in CD4+ CD25+ regulatory T-cell selection. Immunol. Rev..

[bib34] Raefsky E.L., Platanias L.C., Zoumbos N.C., Young N.S. (1985). Studies of interferon as a regulator of hematopoietic cell proliferation. J. Immunol..

[bib35] Redd A.D., Avalos A., Essex M. (2007). Infection of hematopoietic progenitor cells by HIV-1 subtype C, and its association with anemia in southern Africa. Blood.

[bib36] Rouse B.T., Sarangi P.P., Suvas S. (2006). Regulatory T cells in virus infections. Immunol. Rev..

[bib37] Sakaguchi S. (2004). Naturally arising CD4+ regulatory T cells for immunologic self-tolerance and negative control of immune responses. Annu. Rev. Immunol..

[bib38] Shevach E.M. (2006). From vanilla to 28 flavors: Multiple varieties of T regulatory cells. Immunity.

[bib39] Solomou E.E., Rezvani K., Mielke S., Malide D., Keyvanfar K., Visconte V., Kajigaya S., Barrett A.J., Young N.S. (2007). Deficient CD4+ CD25+ FOXP3+ T regulatory cells in acquired aplastic anemia. Blood.

[bib40] Sprent J., Surh C.D., Agus D., Hurd M., Sutton S., Heath W.R. (1994). Profound atrophy of the bone marrow reflecting major histocompatibility complex class II-restricted destruction of stem cells by CD4+ cells. J. Exp. Med..

[bib41] Suffia I.J., Reckling S.K., Piccirillo C.A., Goldszmid R.S., Belkaid Y. (2006). Infected site-restricted Foxp3+ natural regulatory T cells are specific for microbial antigens. J. Exp. Med..

[bib42] Thornton A.M., Shevach E.M. (2000). Suppressor effector function of CD4+CD25+ immunoregulatory T cells is antigen nonspecific. J. Immunol..

[bib43] van Santen H.M., Benoist C., Mathis D. (2004). Number of T reg cells that differentiate does not increase upon encounter of agonist ligand on thymic epithelial cells. J. Exp. Med..

[bib44] von Boehmer H. (2005). Mechanisms of suppression by suppressor T cells. Nat. Immunol..

[bib45] Wang Y., Kissenpfennig A., Mingueneau M., Richelme S., Perrin P., Chevrier S., Genton C., Lucas B., DiSanto J.P., Acha-Orbea H. (2008). Th2 lymphoproliferative disorder of LatY136F mutant mice unfolds independently of TCR-MHC engagement and is insensitive to the action of Foxp3+ regulatory T cells. J. Immunol..

[bib46] Welsh R.M., Selin L.K. (2002). No one is naive: The significance of heterologous T-cell immunity. Nat. Rev. Immunol..

[bib47] Wong J., Obst R., Correia-Neves M., Losyev G., Mathis D., Benoist C. (2007). Adaptation of TCR repertoires to self-peptides in regulatory and nonregulatory CD4+ T cells. J. Immunol..

[bib48] Yamazaki S., Iyoda T., Tarbell K., Olson K., Velinzon K., Inaba K., Steinman R.M. (2003). Direct expansion of functional CD25+ CD4+ regulatory T cells by antigen-processing dendritic cells. J. Exp. Med..

[bib49] Zajac A.J., Blattman J.N., Murali-Krishna K., Sourdive D.J.D., Suresh M., Altman J.D., Ahmed R. (1998). Viral immune evasion due to persistence of activated T cells without effector function. J. Exp. Med..

[bib50] Zhumabekov T., Corbella P., Tolaini M., Kioussis D. (1995). Improved version of a human CD2 minigene based vector for T cell-specific expression in transgenic mice. J. Immunol. Methods.

[bib51] Zinkernagel R.M. (2002). Immunity, immunopathology and vaccines against HIV?. Vaccine.

[bib52] Zinkernagel R.M., Hengartner H. (2001). Regulation of the immune response by antigen. Science.

