# Supplementary material for: Retrovirus-Specificity of Regulatory T Cells Is Neither Present nor Required in Preventing Retrovirus-Induced Bone Marrow Immune Pathology
Source: Immunity. 2008 Nov 14;29(5-2):782–94. doi: 10.1016/j.immuni.2008.09.016 (PMC2631611; doi:10.1016/j.immuni.2008.09.016)
Supplement: Document S1. Supplemental Experimental Procedures and Seven Figures [file mmc1.pdf]

Immunity, Volume *29*

Supplemental Data

Retrovirus-Specificity of Regulatory T Cells

Is Neither Present nor Required in Preventing

Retrovirus-Induced Bone Marrow Immune Pathology

Inês Antunes, Mauro Tolaini, Adrien Kissenpfennig, Michihiro Iwashiro, Kagemasa Kuribayashi, Bernard Malissen, Kim Hasenkrug, and George Kassiotis

Supplemental Experimental Procedures

Assessment of hematopoietic colony-forming cells

Bone marrow cells were suspended and plated in methylcellulose-containing IMDM (MethoCult media, StemCell Technologies, Vancouver, BC, Canada), according to manufacturer's instruction. Burst-forming units-erythroid (BFU-E) were assessed by colony formation on day 8 in media supplemented with erythropoietin. Colony-forming units-granulocyte/macrophage (CFU-GM) were assessed by colony formation on day 6 in media supplemented with SCF, IL-3, IL-6 and GM-CSF.

Bone marrow histology

Histological examination of bone marrow biopsies was carried out by IZVG Pathology, Leeds, UK. Briefly, femurs were isolated form donor mice and decalcified for 24 hours in 15% v/v formic acid/5% v/v formaldehyde solution. Longitudinal sections were prepared, stained with haematoxylin and eosin and photographed under light microscopy.

Dendritic cell pulsing with FV

Dendritic cells were prepared from bone marrow cultures treated with GM-CSF. Cells were pelleted, resuspended in undiluted FV stock (10% w/v spleen homogenate), and incubated at 37°C for 1 hour. Cells were subsequently washed extensively with IMDM to remove the excess FV and used for stimulation of T cells.

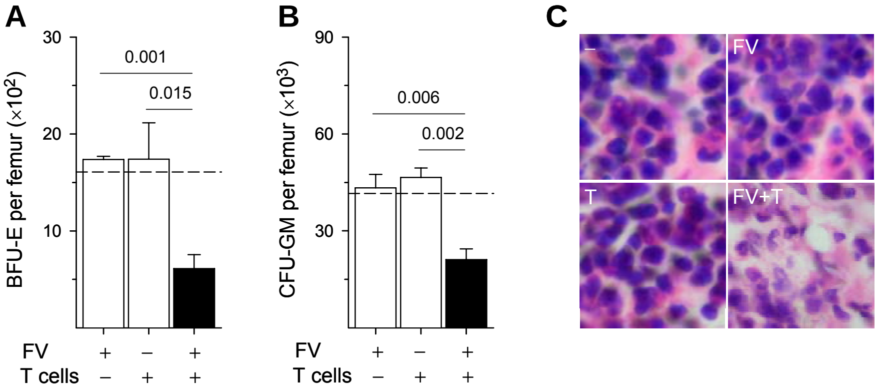

Figure S1. Continuous T helper response to unresolving FV infection causes bone marrow suppression.
(A-B) Numbers of burst-forming units-erythroid (BFU-E) (A) and colony-forming units-granulocyte/macrophage (CFU-GM) (B) in the bone marrow of *Rag1*$^{-/-}$ mice, which were infected with FV, or received EF4.1 TCRβ-transgenic CD4^{+} T cells or both. The dashed line represents the average colony-forming cell number of untreated *Rag1*$^{-/-}$ mice. Values are the mean (±SEM) number of colony-forming cells per femur of 3-5 mice per group on day 21. Numbers within the graphs denote the *P* values.
(C) Haematoxylin and eosin stained femur sections from *Rag1*$^{-/-}$ mice, which were unmanipulated (-) or infected with FV (FV), or received EF4.1 TCRβ-transgenic CD4^{+} T cells (T) or infected with FV and received EF4.1 TCRβ-transgenic CD4^{+} T cells (FV+T). Compact hyperchromatic nuclei (blue) are predominantly normoblasts. Sections are 600x magnified and represent 3-5 mice per group.

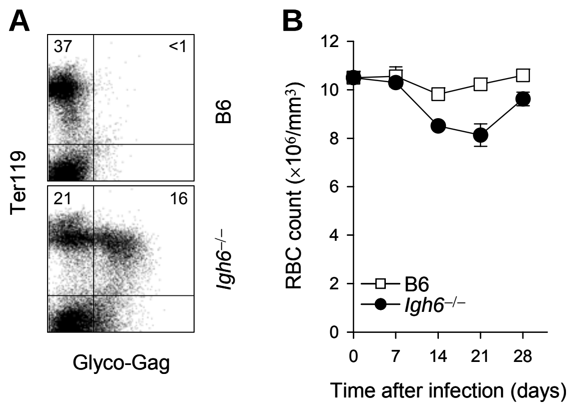

Figure S2. Bone marrow infection and anemia development in B cell-deficient mice.

(A) Percentages of uninfected and FV-infected (glyco-Gag^{+}) Ter119^{+} cells in the bone marrow of wild-type B6 mice (B6) and B cell-deficient (*Igh6*$^{-/-}$) mice 28 days after FV infection. Numbers within the plots represent the percentage of positive cells and are the average of 4-5 mice per group.

(B) Changes in RBC counts in the same groups of mice described in A. Values represent the mean (±SEM) of 4-5 mice per group per time point, analyzed in 2 independent experiments. $P < 0.01$ between 'B6' and '*Igh6*$^{-/-}$' for days 14-21.

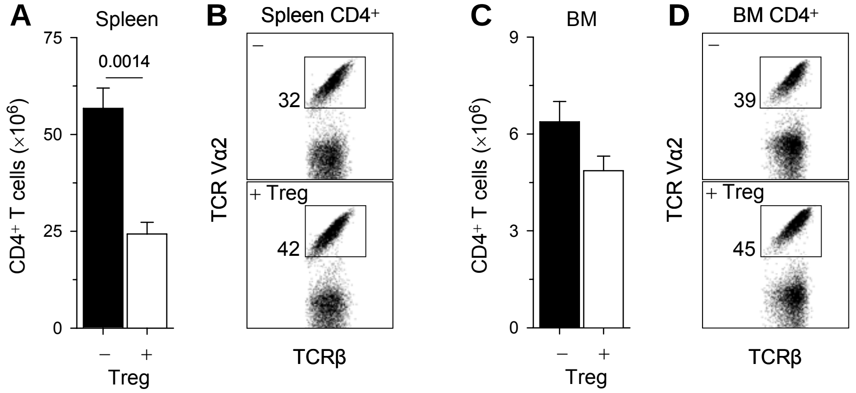

Figure S3. FV-specific EF4.1 TCRβ-transgenic $CD4^+$ T cell expansion in the presence of EF4.1 TCRβ-transgenic Treg cells.
(A-D) Absolute numbers of $CD45.1^+$ EF4.1 TCRβ-transgenic $CD4^+$ T cells (A, C) and percentage of TCR $V\alpha 2^+$ T cells in these cells (B, D), isolated from the spleen (A, B) or the bone marrow (C, D) of FV-infected *Rag1*$^{-/-}$ mice, which received total $CD45.1^+$ EF4.1 TCRβ-transgenic $CD4^+$ T cells, alone (-) or together (+ Treg) with $CD45.2^+$ EF4.1 TCRβ-transgenic Treg cells at 1:1 ratio.

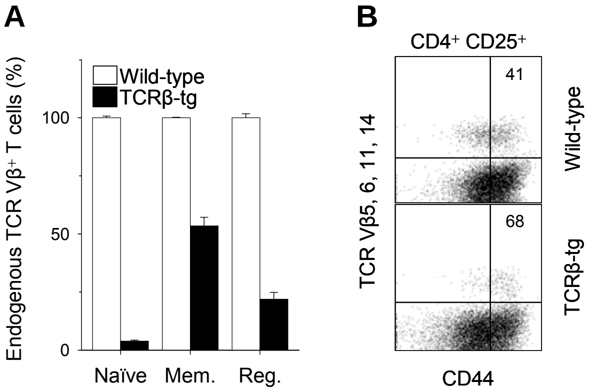

Figure S4. Expression of endogenous TCR Vβ chains in EF4.1 TCRβ-transgenic $CD4^+$ T cell subsets.

(A) The cumulative percentage of T cells expressing TCR Vβ2, Vβ3, Vβ4, Vβ5.1/5.2, Vβ6, Vβ7, Vβ8.1/8.2/8.3, Vβ9, Vβ10b, Vβ11, Vβ12, Vβ13, Vβ14 or Vβ17a, in naïve, memory (mem.) and regulatory (reg.) $CD4^+$ T cells from EF4.1 TCRβ-transgenic (TCRβ-tg) and wild-type littermate control mice (wild-type) is shown. These TCR Vβ chains are used by ~75% of $CD4^+$ T cells in wild-type B6 mice. The sum of measured TCR Vβ expression in wild-type B6 mice was set to 100% and, assuming that allelic exclusion by the transgenic TCRβ chain is similar for the remaining ~25% of endogenous TCR Vβ chains not covered by the panel, the residual expression of endogenous TCR Vβ expression in EF4.1 TCRβ-transgenic $CD4^+$ T cells was then calculated.

(B) CD44 expression by wild-type and EF4.1 TCRβ-transgenic (TCRβ-tg) Treg cells according to expression of endogenous TCR Vβ chain expression. Numbers within the quadrants denote the percentage of T cells which express high levels of CD44 in endogenous TCR Vβ5.1/5.2, Vβ6, Vβ11 or Vβ14-expressing $CD25^+CD4^+$ T cells. Values in A and B are the mean (±SEM) of 3 mice per group from one experiment, representative of 3 similar experiments.

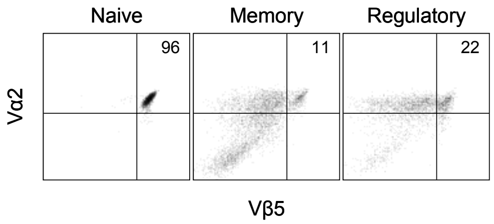

Figure S5. TCR allelic exclusion in OT-II TCR transgenic $CD4^+$ T cell subsets. Expression of OT-II TCRα (Vα2) and TCRβ (Vβ5) transgenes in naïve, memory and regulatory gated $CD4^+$ T cells from OT-II (*Rag1*$^{+/+}$) mice. Numbers within the quadrants depict the percentage of $V\alpha2^+V\beta5^+$ cells and represent the average values from 3 mice.

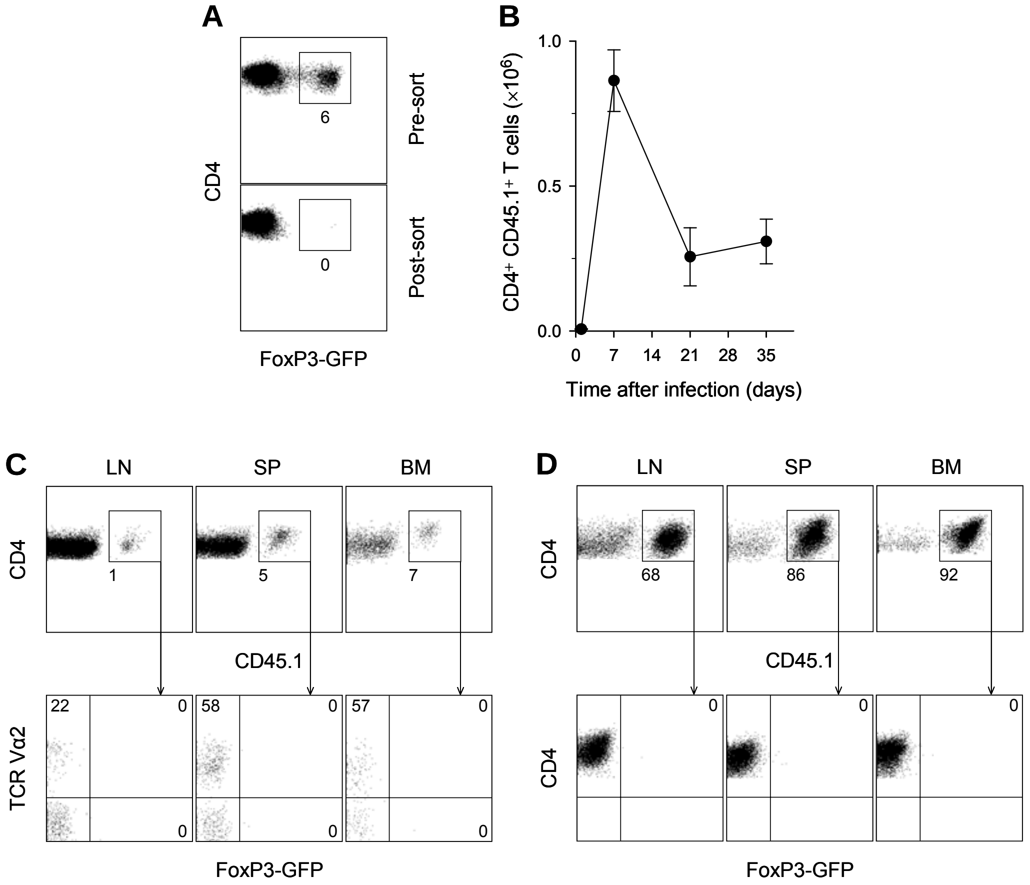

Figure S6. Lack of peripheral conversion of FV-specific EF4.1 TCRβ-transgenic CD4$^+$ T cells into FoxP3-expressing Treg cells.

(A) Flow cytometric example of GFP expression in EF4.1 TCRβ-transgenic *Foxp3^{egfp}* CD4$^+$ T cells before (pre-sort) and after (post-sort) isolation of FoxP3$^-$ (GFP$^-$) T cells.

(B) Absolute number of CD45.1$^+$ CD4$^+$ T cells in the spleen of wild-type B6 recipients of CD45.1$^+$ EF4.1 TCRβ-transgenic *Foxp3^{egfp}* CD4$^+$ T cells over the course of FV infection. Values are the mean (±SEM) of 5-7 mice per time point.

(C) Percentage of FoxP3$^+$ (GFP$^+$) cells in CD45.1$^+$ CD4$^+$ T cells isolated from the lymph nodes (LN), spleen (SP) or bone marrow (BM) of FV-infected wild-type B6 recipients of purified FoxP3$^-$ (GFP$^-$) CD45.1$^+$ EF4.1 TCRβ-transgenic *Foxp3^{egfp}* CD4$^+$ T cells, 35 days after transfer. Numbers within the quadrants depict the percentage of positive cells and are representative of a total of 6 mice analyzed in two separate experiments.

(D) Percentage of FoxP3$^+$ (GFP$^+$) cells in CD45.1$^+$ CD4$^+$ T cells isolated from the lymph nodes (LN), spleen (SP) or bone marrow (BM) of FV-infected *Rag1$^{-/-}$* recipients of purified FoxP3$^-$ (GFP$^-$) CD45.1$^+$ EF4.1 TCRβ-transgenic *Foxp3^{egfp}* CD4$^+$ T cells and CD45.2$^+$ wild-type CD25$^+$ CD4$^+$ Treg cells, at 1:1 ratio, 35 days after transfer. Numbers within the quadrants depict the percentage of positive cells and are representative of a total of 4 mice from a single experiment.

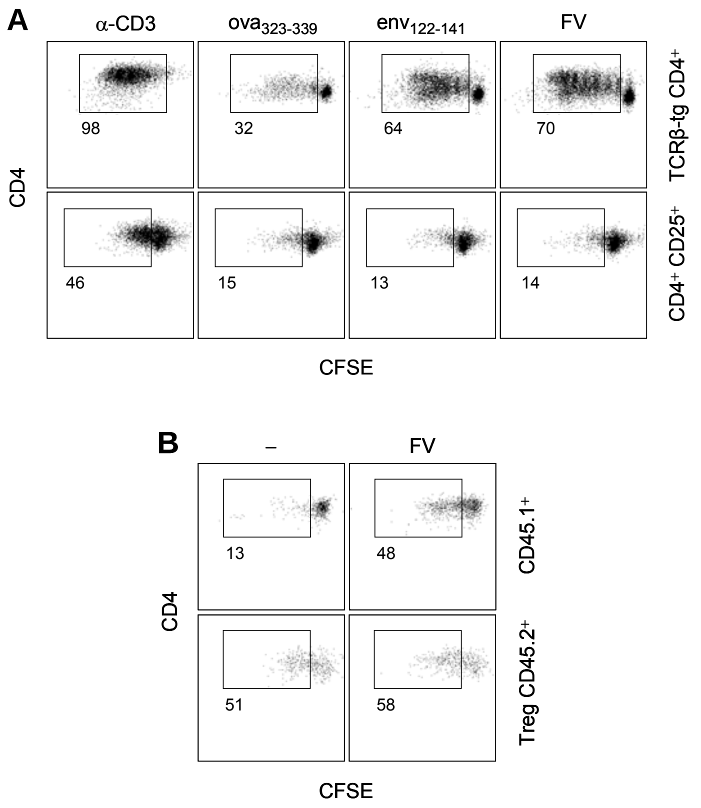

Figure S7. Lack of *in vitro* proliferative response of Treg cells from FV-infected mice to FV antigens.
(A) CFSE dilution profile of $CD4^+$ T cells from virus-naïve EF4.1 TCRβ-transgenic mice (TCRβ-tg $CD4^+$) or $CD25^+$ Treg cells from FV-infected wild-type B6 mice ($CD25^+CD4^+$) 5 days following stimulation by dendritic cells in the presence of anti-CD3, or $ova_{323\text{-}339}$ or $env_{122\text{-}141}$ peptides, or whole FV. Numbers within the plots denote the percentage of divided cells and are representative of 3 mice at day 35 after FV infection. Virus-naïve EF4.1 TCRβ-transgenic $CD4^+$ T cells were used as control for the efficiency of FV-derived epitope presentation by FV-pulsed dendritic cells.
(B) CFSE dilution profile of purified $CD45.1^+$ $CD4^+$ effector T cells ($CD45.1^+$) or $CD45.2^+$ $CD4^+$ Treg cells (Treg $CD45.2^+$) isolated from the bone marrow of FV-infected $Rag1^{-/-}$ recipients of $CD45.1^+$ EF4.1 TCRβ-transgenic $CD4^+$ T cells and $CD45.2^+$ wild-type $CD25^+$ $CD4^+$ Treg cells, 5 days following stimulation by dendritic cells in the absence (-) or the presence of whole FV (FV). Numbers within the plots denote the percentage of divided cells and are representative of 5 mice at day 21 after infection.
